# The complete mitochondrial genome of Eastern paradise fish (*Polynemus dubius*)

**DOI:** 10.1080/23802359.2016.1144094

**Published:** 2016-03-28

**Authors:** Jia Li, Hui Yu, Zhiqiang Ruan, Xingyu Ma, Jimin Zhang, Min Wang, Qiong Shi, Chao Bian

**Affiliations:** a BGI Education Center, University of Chinese Academy of Sciences, Shenzhen, China;; b Shenzhen Key Lab of Marine Genomics, Guangdong Provincial Key Lab of Molecular Breeding in Marine Economic Animals, BGI, Shenzhen, China;; c BGI-Zhenjiang Institute of Hydrobiology, Zhenjiang, China;; d BGI-Zhenjiang Detection Co. Ltd., Zhenjiang, China

**Keywords:** Mitochondrial genome, phylogenetic, *Polynemus dubius*

## Abstract

Eastern paradise fish, *Polynemus dubius* (also called *P. longipectoralis*), lives in rivers of the Southeastern Asia. Although its whole genome is not available, we first obtained its complete mitochondrial genome through next-generation sequencing. The length of its mitochondrial genome is 16 568 bp with 13 protein-coding genes, two rRNA genes, 22 tRNA genes and one control region. The distribution and arrangement of genes in the mitochondrial genome are similar to those of other vertebrates. The GC content is 43.08%, similar to the reported *P. paradiseus*. A phylogenetic tree was constructed to compare with nine related species by using MEGA 6.0. Through phylogenetic analysis, the relationship of species within the *Polynemus* genus is determined.

Eastern paradise fish, *Polynemus dubius* (Motomura 2003), also called *Polynemus longipectoralis*, lives in the Kangsar and Muar rivers (western Malaysia in Malay Peninsula), Musi and Batanghari rivers (southeastern Sumatra, Indonesia), and Sampit and Barito rivers (southern Kalimantan, Indonesia) (Motomura & Tsukawaki 2006). We obtained its samples from the wild (Guangdong Province, China) and stored at China National Genebank (accession no. GZ2014122001). Although its genome is not available, we sequenced its mitochondrial genome using Illumina Hiseq 4000 sequencing platform (Illumina Inc., San Diego, CA) (Tang et al. 2015). Finally, we assembled the complete mitochondrial genome. By utilizing the acquired mitochondrial genome, we can easily distinguish and even protect this precious fish.

Total length of the complete mitochondrial genome of *P. dubius* (accession no. KU199001) is 16 555 bp (which contains 13 protein-coding genes while *nad6* in a reverse orientation), two rRNA genes (12s rRNA with 942 bp and 16s rRNA with 1704 bp in length), 22 tRNA genes and one control region (774 bp). The length of the 22 tRNA genes ranges from 64 bp to 73 bp. However, 10 of the 22 tRNA genes are located in a reverse orientation (Wyman et al. 2004). The start codon of 11 protein-coding genes is ATG, while the rest two genes are starting with GTG and GAC, respectively. Overall, the composition of complete sequence is A (29.91%), T (27.01%), C (27.93%), G (15.15%), and the overall GC-content is 43.08%.

To evaluate accuracy of the assembly and annotation, we downloaded complete mitochondrial genomes of nine reported fish species, including *Eleutheronema tetradactylum*, *Polydactylus plebeius*, *Polydactylus sextarius*, *Polynemus paradiseus*, *Sphyraena barracuda*, *Sphyraena japonica*, *Danio rerio* and *Oryzias latipes* for comparison. We used Muscle Program (vision: v3.8.31; Edgar 2004) to execute the whole genome alignment. Subsequently, we chose the conserved blocks by the online Gblocks (http://phylogeny.lirmm.fr/). A neighbour-joining tree based on the maximum-likelihood method was constructed based on the conserved blocks by using MEGA 6.0 Program (Tamura et al. 2013). Our results demonstrate that the *P. dubius* is much closer to *P. paradiseus* than *P. sextarius* (Figure 1).

**Figure 1.**
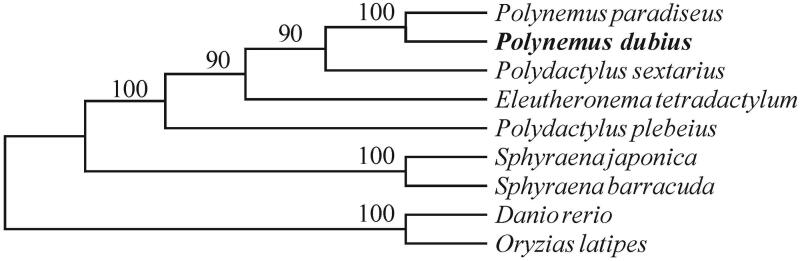
The rectangular phylogenetic tree based on the conserved blocks within mitochondrial genomes of ten related species. The analysis was performed using the MEGA 6.0 software. Accession number: *Eleutheronema tetradactylum* (NC_021620.1), *Polydactylus plebeius* (NC_026235.1), *Polydactylus sextarius* (NC_027088.1), *Polynemus paradiseus* (NC_026236.1), *Sphyraena barracuda* (NC_022484.1), *Sphyraena japonica* (NC_022489.1), *Danio rerio* (NC_002333.2) and *Oryzias latipes* (NC_004387.1)

## References

[CIT0001] Edgar RC. 2004 MUSCLE: multiple sequence alignment with high accuracy and high throughput. Nucleic Acids Res. 32:1792–1797. 15034147 10.1093/nar/gkh340 PMC390337

[CIT0002] Motomura H. 2003 A new species of freshwater threadfin, *Polynemus aquilonaris*, from Indochina, and redescription of Polynemus dubius Bleeker, 1853 (Perciformes: Polynemidae). Ichthyological Res. 50:154–163.

[CIT0003] Motomura H, Tsukawaki S. 2006 New species of the threadfin genus Polynemus (Teleostei: Polynemidae) from the Mekong River basin, Vietnam, with comments on the Mekong species of Polynemus. Raffles Bull Zool. 54:459–464.

[CIT0004] Tamura K, Stecher G, Peterson D, Filipski A, Kumar S. 2013 MEGA6: molecular evolutionary genetics analysis version 6.0. Mol Biol Evol. 30:2725–2729. 24132122 10.1093/molbev/mst197 PMC3840312

[CIT0005] Tang M, Hardman CJ, Ji Y, Meng G, Liu S, Tan M, Yang S, Moss ED, Wang J, Yang C. 2015 High‐throughput monitoring of wild bee diversity and abundance via mitogenomics. Methods Ecol Evol. 6:1034–1043. 27867467 10.1111/2041-210X.12416 PMC5111398

[CIT0006] Wyman SK, Jansen RK, Boore JL. 2004 Automatic annotation of organellar genomes with DOGMA. Bioinformatics. 20:3252–3255. 15180927 10.1093/bioinformatics/bth352

